# P-224. History of penicillin allergy label is associated with adverse outcomes in a matched HIV cohort

**DOI:** 10.1093/ofid/ofaf695.446

**Published:** 2026-01-11

**Authors:** Brayden Seliger, Eric G Sahloff, Joan Duggan

**Affiliations:** University of Toledo, PERRYSBURG, OH; Univ of Toledo, Toledo, OH; University of Toledo Medical Center, Toledo, Ohio

## Abstract

**Background:**

Penicillin allergy labels (PALs) are documented in ∼10% of the U.S. population. People with HIV (PWH) represent a vulnerable population in whom antibiotic stewardship is essential. The impact of PALs on outcomes in PWH has not been well characterized.

**Figure 1:**
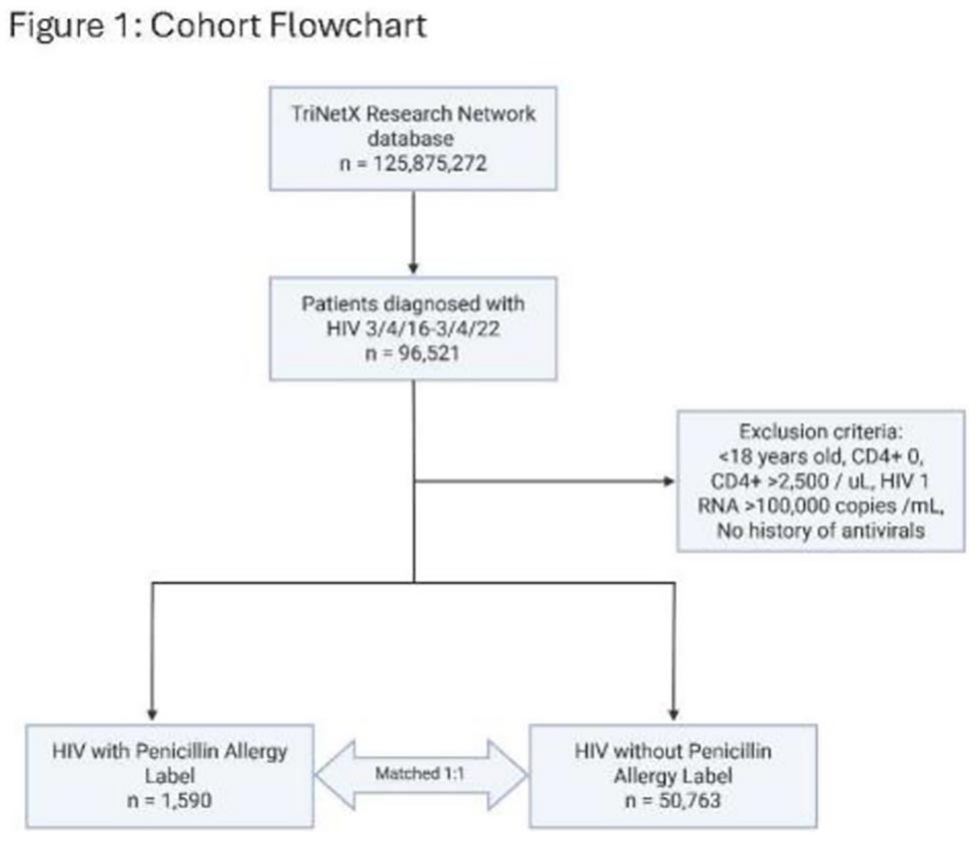
Cohort Flowchart

**Table 1:**
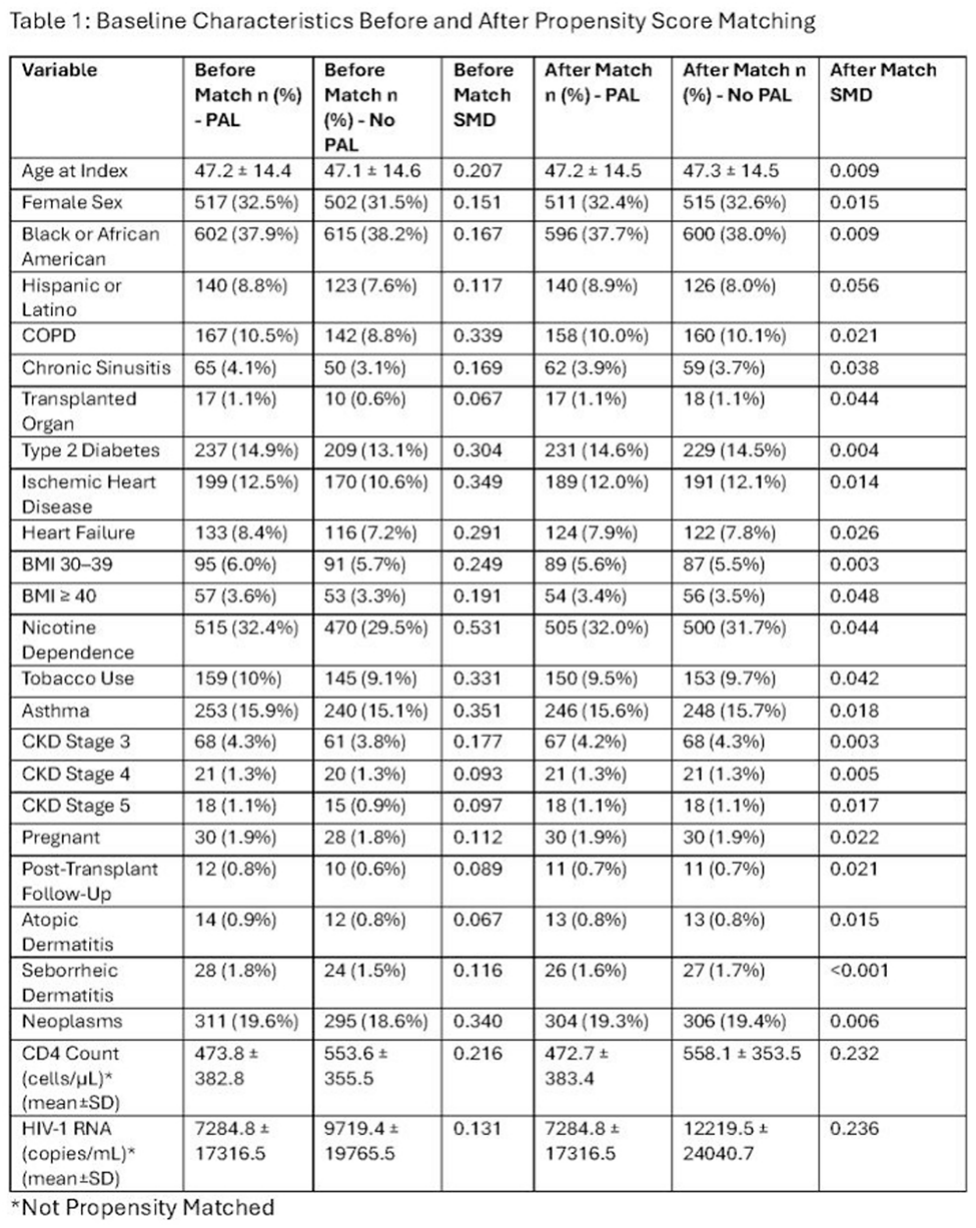
Baseline Characteristics Before and After Propensity Score Matching *Not Propensity Matched

**Methods:**

We conducted a retrospective cohort study using the TriNetX US Collaborative Network (2016–2022) with 3 year outcomes. Adult PWH with a documented PAL were propensity score matched (PSM) 1:1 to those without a PAL based on 23 covariates (n = 1,579 per group) (Figure 1). Outcomes included antibiotic prescribing patterns, rates of infection, inpatient care, critical care, and mortality. Standardized mean differences were used to assess covariate balance. Risk ratios (RR) and 95% confidence intervals (CI) were calculated for all outcomes.

**Figure 2:**
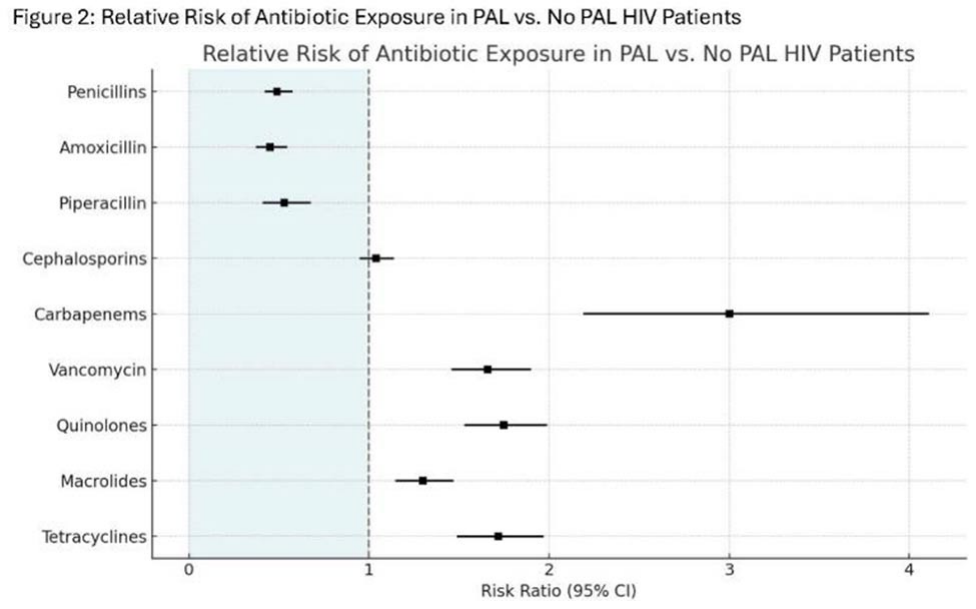
Relative Risk of Antibiotic Exposure in PAL vs. No PAL HIV Patients

**Figure 3:**
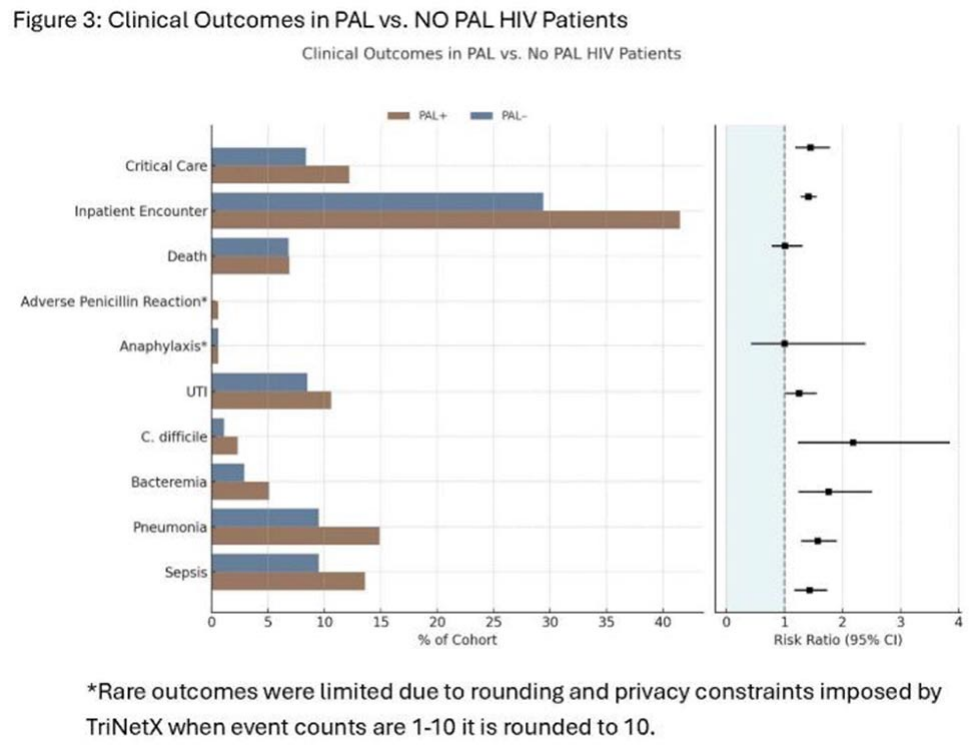
Clinical Outcomes in PAL vs. NO PAL HIV Patients *Rare outcomes were limited due to rounding and privacy constraints imposed by TriNetX when event counts are 1-10 it is rounded to 10.

**Results:**

Baseline characteristics before and after PSM are listed in Table 1: History of PAL was associated with significantly increased use of broad-spectrum antibiotics, including carbapenems (Figure 2). vancomycin, quinolones, macrolides, and tetracyclines. PAL was associated with increased risk of adverse clinical outcomes, including C. difficile infection (2.3% vs 1.1%; RR, 2.18; 95% CI, 1.23–3.85), sepsis (13.6% vs 9.5%; RR, 1.43; 95% CI, 1.17–1.74), bacteremia (5.1% vs 2.9%; RR, 1.76; 95% CI, 1.24–2.51), pneumonia (14.9% vs 9.5%; RR, 1.57; 95% CI, 1.29–1.90), urinary tract infection (10.6% vs 8.5%; RR, 1.25; 95% CI, 1.01–1.56), inpatient admission (41.5% vs 29.4%; RR, 1.41; 95% CI, 1.28–1.55), and critical care services (12.2% vs 8.4%; RR, 1.45; 95% CI, 1.18–1.79) (Figure 3). No significant differences were observed in all-cause mortality (RR, 1.01; 95% CI, 0.78–1.31).

**Conclusion:**

PALs in PWH are associated with increased use of broad-spectrum antibiotics and higher rates of infectious complications and healthcare utilization. These findings underscore the need for widespread allergy delabeling initiatives and targeted stewardship efforts in immunocompromised populations.

**Disclosures:**

All Authors: No reported disclosures

